# Virginia State Dental Association

**Published:** 1879-03

**Authors:** 


### ARTICLE IV.

### *The Virginia State Dental Association.*

The Ninth Annual Session of the Virginia State Dental Association was held in Wilkinson's Hall, in the City of Richmond, commencing Tuesday morning, December 10th, 1878.

At the hour appointed, by reason of the heavy rain falling, it was deemed best to adjourn until 4 o'clock in the afternoon, so as to allow a fuller attendance, at which time the Association was called to order. President J. Hall Moore, of Richmond, in the chair.

The minutes of last meeting were read.

The Secretary read letters from ex-president W. W. H. Thackston, and many others, expressing regrets at not being able to be present to join in annual reunion with their co-laborers in pushing forward the interests and objects of the Association, which are to cultivate the science and art of dentistry.

Dr. Scribner stated that Dr. Norris was detained by illness.

Prof. Harris, of the Baltimore Dental College, and Mr. J. W. Selby, of Baltimore, were introduced to the Association and invited to seats.

The Executive Committee reported the following persons for active membership, all of whom were duly elected:

Dr. J. W. Tucker, of Petersburg; Dr. Henry C. Jones, of Richmond; Dr. Jno. Hartman, of Petersburg; Dr. Jno. N. Webster, of Norfolk; Dr. Edgar Hartman, of Richmond.

Prof. Harris was elected to honorary membership.

Executive Committee reported that homes had been provided for all that had applied, also that arrangements had been made for clinics.

On motion of Dr. Keesee, the annual address of the President was read, and on motion of Dr. Scribner the address was received and referred to a committee. Drs. Scribner, Cowardin and Chewning, were appointed as the committee, with instructions to report as early as possible.

Dr. Thompson moved that the hours from 9 to 11 be appointed for clinics; 11 to 2 for morning session ; 4 to 7 for afternoon session ; which were adopted.

Prof. Harris and Dr. Cowardin were appointed to conduct the clinics for the following morning, after which, on motion of Dr. Wood, the Association adjourned.

WEDNESDAY MORNING SESSION.

The Association was called to order at 11 o'clock, after having attended the clinic at Dr. Wood's office, conducted by Prof. Harris.

President Moore in the chair. The minutes were read and approved.

Dr. T. S. Waters, of Baltimore, was introduced and invited to a seat.

On the call for reports from committees, the Publication Committee reported that after consultation, it was deemed best not to have printed the 2000 copies of the Dental Bill to regulate the practice of dentistry in the State, as was ordered at the last session.

Report received and passed.

Indulgence was granted Committee on Operative Dentistry until further time to report, and on motion, Prof. Harris was added to the Committee.

Prof. Hodgkin, of the Committee on Mechanical Dentistry, not being present, on account of sickness, on motion the subject was taken up. Dr. Scribner opening the discussion by asking the opinion of those present concerning the use of celluloid. He always heard of his failures, but the successful patients, being satisfied, never return to report. His practice was confined almost exclusively to young patients, and so had but little mechanical dentistry to do. He asked Prof. Harris what was the general advice given students at the College. To which the Professor replied that they did not advise the use of celluloid. In duty to students they taught them the use of all materials, but the young practitioner could not afford to risk his reputation by the use of a material that will warp, and will often disintegrate after being worn twelve months, and is, consequently, so unreliable. It might suit plain, but seldom gum teeth; it would undergo a molecular change; turn dark and soften on the surface, and, in the end, would finally give way; only a few maintained their consistency. In some cases, could scarcely tell what it was; would not even advise its use for temporary plates. On the other hand, the rubber would stand for years, and, he thought, was easier to work.

Dr. Waters' experience did not agree with Prof. Harris'. He preferred the celluloid to rubber; thought it saved time because, if the rubber was at all hastened, it would be hard and brittle. He worked the celluloid in vapor of glycerine, and after getting the plate down, fifteen minutes afterwards, dipped in cold water and cooled thoroughly before removing pressure. Silver and celluloid worked admirably together, but as the silver corroded he could only advise it on account of its cheapness.

Dr. Thompson said he had worked celluloid for four or five years and had found it to be all that he wanted; had no inflammation of the mucous membrane, and never had a gum block break; worked it with steam, and decidedly gave it the preference over rubber, but took care to let it stand as long as possible to cool off.

Dr. Mercer found it to stand equally as well as rubber.

Prof. Harris stated that, with all due respect to the opinion of those who had spoken, his mind was not changed, as he had yet to see the first plate that had not changed in color after being worn a few months. He had even known them to change in twenty-four hours, and the only thing that could at all be said in favor of celluloid was that it was a rather better conductor than rubber. He always advised the patient to remove a rubber plate from the mouth at night, and thus tendency to inflammation was relieved. The material was not always to be blamed for the soreness of the mouth, as some patients kept their dentures in a less cleanly condition than they should.

Dr. Chewning had made extensive inquiries in his city and had yet to find the first one who was square with Rubber Company who did not prefer rubber.

Dr. Waters thought differently, and advised never to polish a celluloid plate beyond the use of sand paper.

Dr. Mercer said he was not in the clutches of the Rubber Company, but yet gave celluloid the preference.

Dr. Keesee said that he was very sorry that his experience in the use of celluloid had compelled him almost entirely to abandon it. He found no trouble in working it, and obtained accurate fits and all that was, perhaps, desirable in the outstart, but yet found that in a very short time the plates would turn most miserably dark (thus presenting a very unsightly appearance where the gum would show,) and had found them to wear so much that in a few months the teeth could, by slight pressure, be shoved from their place, and hence, for the welfare of his patients, he had gone back to the use of rubber.

Dr. Cowardin did not think celluloid was going to give satisfaction in the long run. Did not believe that any of the substances now in use for dental plates, if well fitted to the mouth, would give rise to inflammation unless there is a predisposition to inflammation, which arises from some derangement of the alimentary canal.

Dr. Moore asked if discoloration did not also occur with rubber. To which Prof. Harris replied, yes; but that it was only a stain on the surface, and not a change of plate itself, as was the case with celluloid.

Dr. Moore did not think that celluloid was as durable as rubber, but all mechanical work was *so cheap* that patients ought not to complain. No dentist ought to guarantee a plate to last forever. He used celluloid with satisfaction, and would much prefer it in his own mouth, as he thought it more agreeable. Even gold would produce inflammation, and no mouth *could* be healthy where the plate was worn day and night, and not in addition kept perfectly clean.

Dr. R. Finley Hunt, of Washington, was introduced and invited to participate. He agreed that celluloid was, perhaps, more pleasant to wear, but there was not the certainty of obtaining as favorable results as by the use of rubber. If a *good* plate of celluloid was obtained, he thought it preferable. He still continued the use of dry heat, and had no trouble in warping. Being allowed to digress, he spoke at length upon the legal aspect of the case of the Rubber Company versus the dentists.

On motion the subject of mechanical dentistry was passed, and the Association adjourned to meet at 4 o'clock.

Afternoon Session.

Dr. T. S. Waters was elected an honorary member.

Dr. Chewning, from Committee on Dental Literature and Education, presented a lengthy report, prefacing his remarks by stating that the report was written before his connection with the Baltimore Dental College.

Report was received and discussed at length, especially by Dr. Wood, who felt much gratified in hearing such a report. He believed in specialties and that medical colleges were not qualified to teach dentistry, together with everything else.

Dr. Scribner moved a vote of thanks to Dr. Chewning, which was adopted, and on motion the subject was passed.

Dr. N. M. Burkholder, from Committee on Dental Pathology, presented a most welcome report, as did Dr. Thompson, from the same Committee. The reports were received and discussed by Drs. Hunt, Cowardin and Wood, until the hour of adjournment.

Thursday Morning Session.

The minutes of last meeting were read and approved. The subject of Dental Pathology was resumed and occupied the greater portion of the morning session, a synopsis of which only could be given, which would not do justice to the great interest manifested and participated in by so many members present. Quite reluctantly the subject was passed, and, on motion of Dr. Scribner, the election of officers was ordered as the first business to claim attention of the afternoon session.

Dr. Scribner next addressed the Association on the subject of Plastic Fillings *versus* Gold Fillings, this new departure feature being the one that seemed to disturb the dental profession most at present. He related that a few years or possibly months ago, some of the leading members would only use gold, and never a plastic filling, but for his own part he always found that he had to use discretion.

Dr. Hunt deprecated extremes; had to often adapt ourselves to the pecuniary circumstances of our patients. Did not like exactly the antagonism of plastic filling *versus* gold. Amalgam, he thought, had its many advantages, especially that of producing the hardness so often found beneath a filling. Thought the same care needed for plastic as for gold fillings. For temporary filling he preferred the osteoplastic to gutta percha. Dr. Cowardin had found tin preferable for buccal cavities.

Prof. Harris thought the failures resulted from not always understanding the nature of the case. If possible, he preferred gold, but often excess of saliva would govern what material to use. Did not agree with Prof. Flagg in his new departure theory. Thought all should study thoroughly the nature of gold. Regardless of material, we all had our failures to lament. He was an advocate of using the very softest preparation of gold, but at the same time also was eclectic in choice of materials and appliances. Did not like os artificial for approximal cavities, but preferred gutta- percha—in the use of which he always tried to avoid an excess of material. In a near approach to nerve he used the softest gutta-percha, never wishing to have anything the least escharotic.

Dr. Cowardin did not find os-artificial to suit for cavities with even nearly exposed nerves.

Dr. Scribner had found much service from the use of os-artificial.

Dr. Jones had much pain in the use, but it soon became comfortable.

Dr. Smith spoke of his experience being favorable; using, in connection, a small piece of rubber dam.

Dr. Chewning liked for such purpose Weston's non-irritant cement.

Dr. Thompson said the pain, he thought, could be avoided by using creosote with the oxide of zinc, and capping this with the oxychloride of zinc.

Dr. Keesee had used Dr. Thompson's method, in addition, using a few folds of bibulous paper moistened with creosote just over the nerve, then following up with the other preparation.

As Prof. Harris desired as little irritation as possible, he sometimes used a solution of gutta percha. He always gave the nerve a chance before he would destroy it. Had, at times, found the restoration complete, but it would take from eighteen months to three years; he would never insert a permanent filling at once. Did not like the too free use of gold in the front teeth for children, but preferred, whilst the teeth were so vascular, a plastic filling of gutta percha stopping, or its equivalent. Had experimented with the shrinkage of amalgam and found it extremely difficult to introduce a perfect amalgam filling. If used perfectly dry, it was very difficult to work satisfactorily, nevertheless, it was our duty to persevere.

On motion, the regular order of business was suspended to allow Dr. Hunt to give in detail the legal status of the Goodyear Dental Vulcanite Company *versus* the Dentists.

After which Dr. Thompson offered the following resolution, which was adopted:

*Resolved,* That the Virginia State Dental Association sympathize with Dr. R. Finley Hunt in his efforts to set aside the patent for the use of vulcanite, now claimed by the Goodyear Dental Vulcanite Company, and recommend that the dentists throughout the State render materisl aid to Dr. Hunt in his efforts to set aside the patent.

### Afternoon Session.

Association called to order at 4 o'clock.

According to previous order, the Association proceeded to ballot for officers for the ensuing year which resulted in the election of:

Dr. J. Hall Moore, of Richmond, President ; Dr. J. W. Scribner, of Charlottesville, 1st Vice President: Dr. S. H. Henkel, of Staunton, 2d Vice President; Dr. A. C. Jones, of Richmond, 3d Vice President; Dr. J. F. Thompson, of Fredericksburg, Treasurer ; Dr. Geo. F. Keesee, of Richmond, Recording Secretary; Dr. L. M. Cowardin, of Richmond, Corresponding Secretary; Dr. Geo. B. Steel, of Richmond, Reporting Secretary ; Judd B. Wood, of Richmond ; W. W. H. Thackston, of Farmville ; W. E. Morris, of Charlottesville, Executive Committee.

Dr. Cowardin moved that further discussion of the subject of Operative Dentistry be dispensed with, which was adopted.

On motion of Dr Scribner, the regular order of business was dispensed with so as to allow the selection of place for holding the next Annual Session of the Association, and Charlottesville was selected; and on motion of Dr. Cowardin, it was determined that when the Association adjourned it adjourn to meet on the 3d Tuesday in August, 1879.

On motion of Dr. Steel the Committee on Dental Physiology was excused and subject passed, as also was that on Dental Appliances.

The Committee on President's address presented their report and asked that the following subjects, touched upon in the address, be more fully discussed:

1st. That part referring to the cultivation of a brotherly feeling between the members of the profession.

2d. That part referring to the Dental Colleges and the duty of the Association to help them to do the work of turning out well qualified men.

3d. That part referring to membership in the Association.

Which subjects were discussed at length.

Dr. Wood offered the following amendment to the Code of Ethics:

ARTICLE. 11—Sec. 3d. It is unprofessional to advertise by other means than a card, giving name, title and address. Which he asked to be laid over until next session of the Association.

Dr. Scribner offered the following resolution, which was adopted:

*Resolved*, That a committee of three be appointed whose duty it shall be to have two thousand copies of the Dental Bill printed and send the same, together with Dr. Thackston's Address, to the dentists in the State, with a memorial for the signature of the public, to be returned to said Committee to lay before our Legislature at its next session, in December, 1879.

A Committee was appointed, consisting of Drs. Thackston and Moore, those two to select the third.

The Association then adjourned to meet in Charlottesville, on the 3d Tuesday in August, 1879.—*Dental Miscellany.*

